# When one size doesn’t fit all: aciclovir dosing and the obesity challenge

**DOI:** 10.1136/bmjpo-2025-004237

**Published:** 2026-06-17

**Authors:** Maria Solovyeva, Calandra Feather, Nicholas Appelbaum, Elliott Gordon

**Affiliations:** 1 School of Clinical Medicine, University of Cambridge, Cambridge, UK; 2 Dosium Holdings Ltd, London, UK

**Keywords:** Child Health, Pharmacology

 The global rise in childhood obesity poses major challenges for safe prescribing. Conventional weight-based or body surface area (BSA)-based dosing guidelines often fail to account for differences in body composition, potentially leading to underdosing or toxicity at the extremes of weight centiles. Pharmacovigilance data on aciclovir from the Medicines and Healthcare products Regulatory Agency include >100 paediatric adverse reaction reports since 2015, highlighting an ongoing burden of treatment-related harm.^1^ The British National Formulary for Children (BNFC) recommends weight-based aciclovir dosing up to 3 months and between 12 and 17 years, while BSA-based dosing is used for those aged 3 months to 11 years.^2^ For weight-based dosing, the Neonatal and Paediatric Pharmacy Group advises the use of ideal body weight (IBW) for children >98th percentile weight-for-age and sex. Although this approach may lead to clinically implausible dose reductions at arbitrary thresholds,^3^ it serves to moderate exponential dosing patterns. Alarmingly, no such adjustment is proposed for BSA-based calculations, whereby dosing is derived directly from total body weight (TBW). Aciclovir’s volume of distribution correlates more closely with IBW and total body water than TBW, meaning that dosing based on TBW in children with obesity could exceed pharmacokinetically appropriate levels. In addition, the abrupt transition from BSA-based to weight-based calculations at age 12 results in a sharp dosing drop.

These challenges highlight the clinical need for more nuanced, physiology-informed dosing strategies. We propose a centile-sensitive model using adjusted body weight (AdjBW) and a blending function to improve consistency and safety of aciclovir prescribing.

To characterise current prescribing practices, a series of aciclovir dose-weight-age visualisations was developed using BNFC guidance and UK-WHO growth standard LMS datasets (boys aged 0–17 years, at 50th height centile) via Python and Plotly.^4^ *BSA* was calculated using the Boyd formula below, where *W*=actual body weight (g):^5^


$$BSA=4.688\;\cdot \;W^{\left(0.8168-0.0154\;\cdot \;logW\right)}$$

Two approaches were subsequently investigated to address the incongruent dose transitions.

First, AdjBW was used for children above the 50th centile weight-for-age, as a substitution for actual weight in BSA calculations as a point of comparison. *IBW*, representing 50th centile BMI-for-age, was used for this calculation:^6^


AdjBW=IBW+0.35 (Actual−IBW)

Second, we applied linear interpolation between BSA-based dosing and mg/kg dosing across ages 12–17 to demonstrate the theoretical solution to the abrupt dose drop:


$$Dose_{blend}=\left(1-w\right)\cdot \;Dose_{BSA}+w\;\cdot \;Dose_{kg}$$

Where:

- ${Dose}_{BSA}$ is the BSA dose calculated using AdjBW (250 mg/m^2^).
- ${Dose}_{kg}$ is the dose using the weight-based regimen (5 mg/kg).
- $w=\frac{Age-12}{5}$ is a linear weight factor increasing from 0 at age 12 to 1 at age 17.

**Figure 1:**
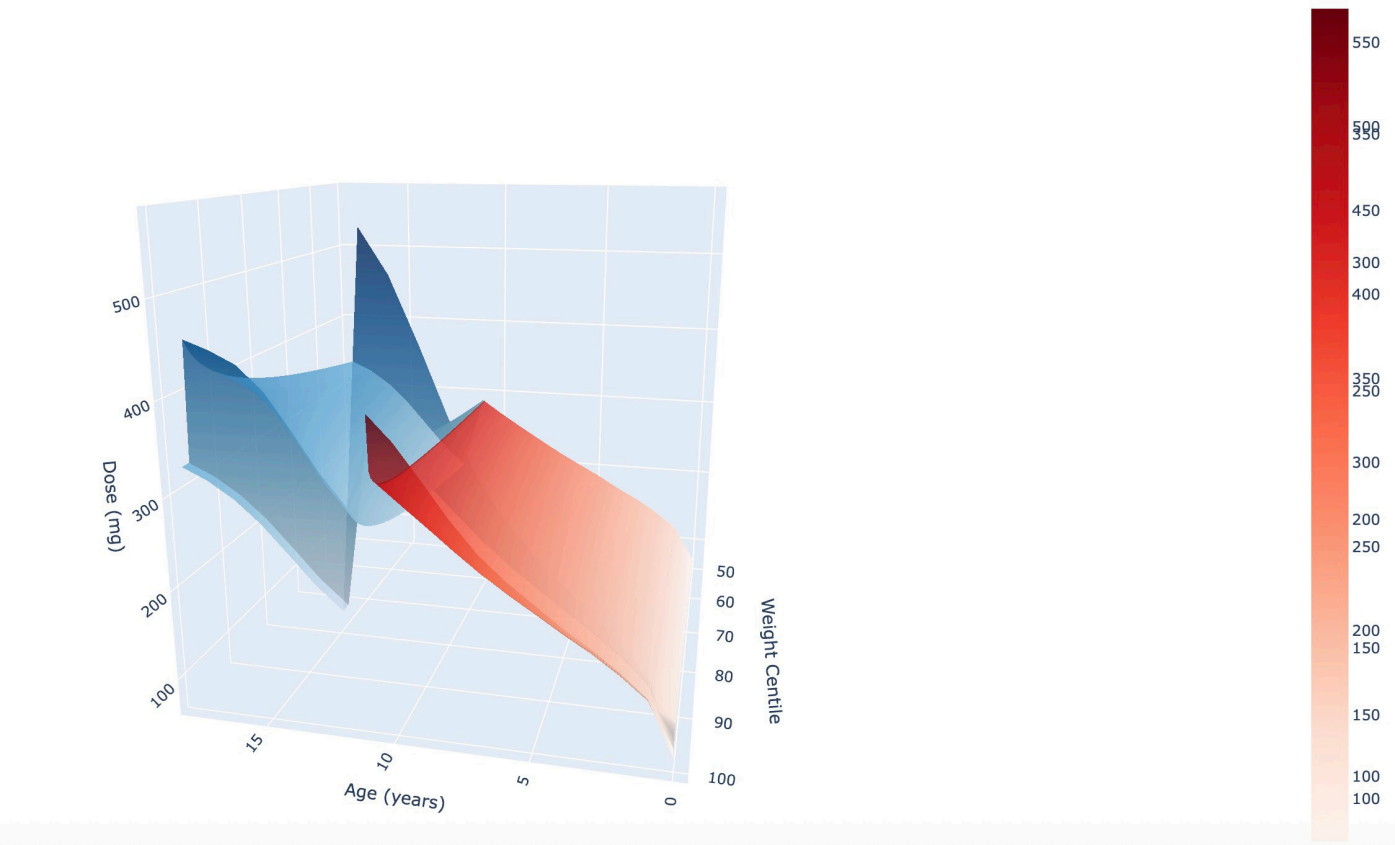
Aciclovir dose (mg) curves for boys aged 0–17 years, across 50th–99.99th weight centiles, comparing original BNFC-approved dosing (blue) and AdjBW-based BSA dosing (red) up to 12 years of age. AdjBW, adjusted body weight; BNFC, British National Formulary for Children; BSA, body surface area.

The blue plane in figure 1 represents aciclovir dosing for boys aged 0–17 years and 50th−99.99th weight centiles based on BNFC guidance. Doses reach as high as 570 mg for 99.99th centile for an 11-year-old, before plunging down to 191.9 mg overnight when they turn 12 due to the use of IBW for mg/kg calculations. The red plane, for comparison, demonstrates the use of AdjBW-BSA as a theoretical alternative that more closely approximates the pharmacokinetically relevant lean compartment and may mitigate overdosing at higher weight centiles, bringing the dose of an 11-year-old at the 99.99th centile to 398.8 mg, a 30% decrease when compared with the original regimen.

**Figure 2:**
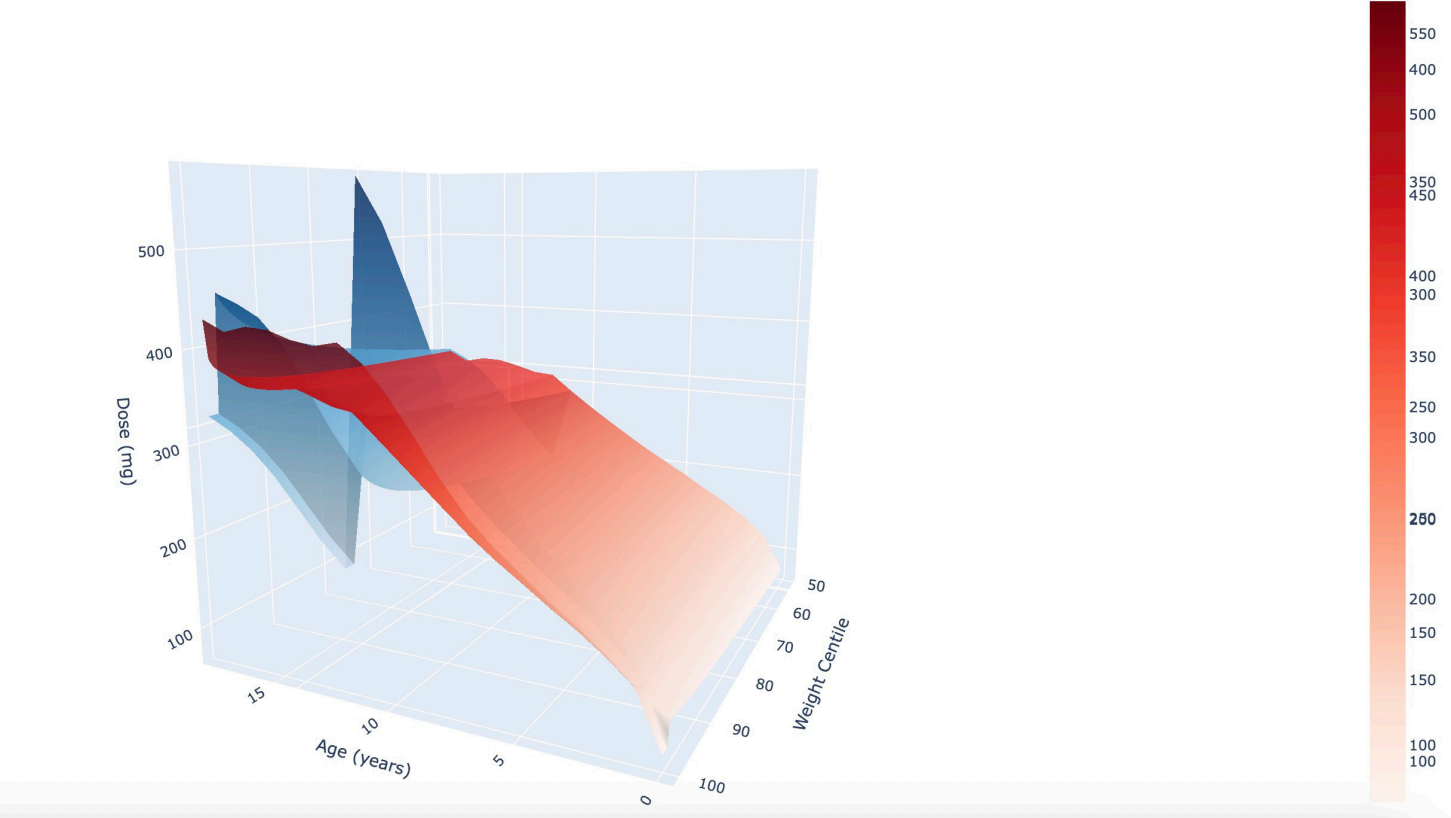
Aciclovir dose (mg) curves for boys aged 0–17 years, across 50th–99.99th weight centiles, comparing original BNFC-approved dosing (blue) and AdjBW-based BSA dosing (red) with further use of blend formula to reach weight-based dosing at age 17. AdjBW, adjusted body weight; BNFC, British National Formulary for Children; BSA, body surface area.

Nonetheless, the abrupt switch of dosing regimens at age 12 remains. The use of the blend formula in figure 2 eliminates clinically incongruent transition to mg/kg dosing, while maintaining dose adjustment at higher weight centiles with AdjBW use.

This analysis demonstrates that using AdjBW in BSA-based dosing offers a substantial improvement over current methods at higher weight centiles where incongruent dose calculations are seen. Albeit not easily translatable into manual prescribing practice due to mathematical complexity, the blended BSA-to-weight-based dosing function demonstrates the continuity and logically sound dosing transitions we should be striving for in the age of digitalisation.^7^ In the meantime, a more pragmatic approach such as a step-down calculation from AdjBSA to weight-based doses could be considered.

We acknowledge that this is a theoretical visualisation based on male anthropometric data at the 50th height centile and it has not been validated against pharmacokinetic or clinical outcome data. In practice, BSA is calculated using individual height and weight and the AdjBW substitution applies irrespective of sex or height; however, the model would benefit from evaluation across both sexes and height standard deviation scores to improve generalisability. Sensitivity analyses of alternative IBW and BSA calculations as well as different obesity-related transition thresholds would also be valuable here. Factors such as renal function, indication and route of administration require independent clinical consideration and sit alongside our proposed anthropometric approach.

Regardless, the rising prevalence of childhood obesity should prompt reconsideration of the current dosing assumptions and further research beyond aciclovir to mitigate the risk of harm in an already vulnerable population.
