# Supplementary material for: When one size doesn’t fit all: aciclovir dosing and the obesity challenge
Source: BMJ Paediatr Open. 2026 Jun 17;10(1):e004237. doi: 10.1136/bmjpo-2025-004237 (PMC13289368; doi:10.1136/bmjpo-2025-004237)
Supplement: Supplementary file 1 [file bmjpo-2025-004237.reviewer_comments.pdf]

PEER REVIEW HISTORY

BMJ Paediatrics Open publishes all reviews undertaken for accepted manuscripts. Reviewers are asked to complete a checklist review form and are provided with free text boxes to elaborate on their assessment. These free text comments are reproduced below.

ARTICLE DETAILS

**TITLE (PROVISIONAL)**	When One Size Doesn't Fit All: Aciclovir Dosing and the Obesity Challenge
**AUTHORS**	Solovyeva, Maria<br>Feather, Calandra<br>Appelbaum, Nicholas<br>Gordon, Elliott

VERSION 1 – REVIEW

**REVIEWER NAME**	Sutherland, Adam
**REVIEWER AFFILIATION**	University of Bradford, School of Pharmacy and Medical Sciences
**REVIEWER CONFLICT OF INTEREST**	No Conflict of Interest
**AI DISCLOSURE**	No, I have not used AI
**DATE REVIEW RETURNED**	19-Nov-2025

**GENERAL COMMENTS**	Thanks very much for the first look at this letter. Really interesting subject and you're really showing us how we can leverage data to understand some of our more challenging clinical conundrums<br>1) Can you give any insight into the burden of harm associated with aciclovir dosing in the paediatric population as a whole? This would help create a sense of urgency and a target for intervention<br>2) Page 1, Line 21 - the NPPG is the Neonatal and Paediatric Pharmacy Group.<br>3) How does your model relate to the use of the UKCCSG surface area bounds in the BNFc which (in practice) is what we all use to estimate BSA in our patients.<br>4) Also, how does the use of Mosteller or DuBois formulae for BSA estimation affect your outcomes? This is important because these formulae are those incorporated in several off-the-shelf EPR systems.<br>5) In your concluding paragraph you state that using AdjBW mitigates "dose inflation" - this reads like you're asserting that doses using BSA are higher than they should be? Perhaps the weight based doses are too low? What is the PK/dose-response relationship for aciclovir in uncomplicate HSV infection? I think this background is what's missing earlier in the letter.<br>6) Your final paragraph about urgent reconsideration of existing guidelines and reducing avoidable harm isn't really supported with this single example. I think you have a reasonable call-to-action around re-evaluating dosing assumptions, but this requires further research.

**REVIEWER NAME**	Wu, Yue-E
**REVIEWER AFFILIATION**	Shandong University
**REVIEWER CONFLICT OF INTEREST**	No Conflict of Interest

**AI DISCLOSURE**	No, I have not used AI
**DATE REVIEW RETURNED**	01-Apr-2026

**GENERAL COMMENTS**	Overall, the article is concise and clinically relevant, and it raises an important issue regarding aciclovir dosing in children with obesity. However, I think there are still several points that need further clarification. My main concerns are as follows.<br><br>Major concerns<br>1. The current analysis appears to be based only on boys aged 0–17 years at the 50th height centile. Could the authors further discuss how generalisable these findings are to girls and to children with heights substantially different from the 50th centile?<br>2. This work is essentially a guideline-based visualisation and theoretical modelling study, rather than a PK/PD or clinical outcome study. Is there any evidence that the proposed AdjBW-based BSA dosing would maintain antiviral efficacy while reducing the risk of overdosing in children with obesity?<br>3. The authors propose using AdjBW for children above the 50th centile weight-for-age. Why was the 50th centile selected as the threshold? Would it be more clinically appropriate to use an obesity-related threshold instead, such as a higher weight centile or BMI-based definition?<br>4. The manuscript uses the formula AdjBW = IBW + 0.35 × (Actual − IBW). Could the authors explain the rationale for choosing the 0.35 coefficient in this paediatric aciclovir context, and whether this coefficient has been validated for antiviral dosing in children?<br>5. The authors define IBW as the 50th centile BMI-for-age for the AdjBW calculation. Could they further justify why this particular definition of IBW was chosen, and whether other definitions of IBW might materially change the proposed dosing curves?<br>6. To address the abrupt dose drop at age 12, the authors apply linear interpolation between BSA-based dosing and 5 mg/kg dosing from age 12 to 17 years. Why was a linear transition chosen, and why does the transition end specifically at 17 years? Would other transition functions or age ranges be more physiologically plausible?<br>7. The manuscript argues that the current BNFC approach leads to an abrupt drop from 570 mg at age 11 to 191.9 mg at age 12 in a very high weight-centile child, and that the proposed approach reduces the 11-year-old dose to 398.8 mg. However, how should readers interpret these numerical differences clinically, given that there are no linked exposure targets, toxicity thresholds, or virological outcomes presented?<br>8. Aciclovir exposure is influenced not only by body size, but also by other factors such as renal function, indication, and route of administration. Since the present analysis focuses mainly on anthropometric dosing rules, could the authors comment on how these additional determinants might affect the clinical applicability of the proposed model?<br><br>Minor comments<br>9. The background states that conventional weight-based or BSA-based dosing may lead to underdosing or toxicity at the extremes of weight centiles. It would strengthen the letter if the authors could provide more direct references supporting this statement specifically in paediatric obesity or in aciclovir prescribing.<br>10. Some of the mathematical expressions in the proof version appear unclear or incompletely rendered, including the Boyd formula and the dose blending equation. I would suggest carefully checking the equation formatting in the final version. In addition, the phrase "AdjBW-base BSA dosing" in the figure legend may need correction to "AdjBW-based BSA dosing."

VERSION 1 – AUTHOR RESPONSE

Reviewer: 1
Dr. Adam Sutherland, University of Bradford
Comments to the Author
Thanks very much for the first look at this letter. Really interesting subject and you're really showing us how we can leverage data to understand some of our more challenging clinical conundrums

1) Can you give any insight into the burden of harm associated with aciclovir dosing in the paediatric population as a whole? This would help create a sense of urgency and a target for intervention.
Response: Thank you for this important point. We have included reference to pharmacovigilance data from the MHRA Yellow Card Scheme in the first paragraph to highlight reported adverse drug reactions to aciclovir in children, however, we do acknowledge that these are not necessarily attributable to the dosing discontinuity described. We used this as a signal of ongoing treatment-related harm in paediatric practice, and as a rationale to explore opportunities for optimisation in dosing strategies, particularly in populations at higher risk of disproportionate exposure.
Changes made: Reference and comment on the pharmacovigilance data from the MHRA Yellow Card Scheme in paragraph 1.

2) Page 1, Line 21 - the NPPG is the Neonatal and Paediatric Pharmacy Group.
Response: Corrected, thank you.
Changes made: edited wording of NPPG abbreviation in paragraph 1.

3) How does your model relate to the use of the UKCCSG surface area bounds in the BNFc which (in practice) is what we all use to estimate BSA in our patients.
Response: We appreciate this is a theoretical model that uses exact BSA calculations, whereas UKCSG surface area banding is often used as a pragmatic bedside tool instead. However, these bands are inherently limited, particularly as they often only extend up to around 40 kg or the 90th centile in certain age groups e.g. 10-year-olds. In addition, UKCCSG banding is based on total body weight and doesn't distinguish lean from obese children at the same weight. In contrast, our model incorporates adjusted body weight (AdjBW) upstream, generating a lower and more physiologically appropriate BSA in children with obesity, whilst converging with the standard approach at normal weight centiles.
Our aim was specifically to investigate children at the extremes of weight centiles, a population that is not well represented within these banding systems. By using BSA as a continuous variable via the Boyd formula, we were able to perform a more granular analysis across the full distribution of weight and age, rather than being constrained by predefined categories. This approach allows us to identify age groups and weight centiles where dosing outputs become physiologically implausible or inconsistent, which may be obscured when using banded approximations.
Importantly, this methodology also reflected the increasing use of electronic prescribing, where BSA is likely to be calculated automatically and the discrepancies that may emerge in digital prescribing environments.
Changes made: A note on alternative BSA calculations was included in the limitations of the penultimate paragraph.

4) Also, how does the use of Mosteller or DuBois formulae for BSA estimation affect your outcomes? This is important because these formulae are those incorporated in several off-the-shelf EPR systems.
Response: This study, as a theoretical visualisation, utilised the Boyd formula for BSA estimation, in line with its use in the BNFC. However, we acknowledge that further work is needed to evaluate how the use of alternative BSA formulae may influence these findings.
Changes made: A note on alternative BSA calculations was included in the limitations of the penultimate paragraph.

5) In your concluding paragraph you state that using AdjBW mitigates "dose inflation" - this reads like you're asserting that doses using BSA are higher than they should be? Perhaps the weight-based doses are too low? What is the PK/dose-response relationship for aciclovir in uncomplicate HSV infection? I think this background is what's missing earlier in the letter.
Response: Thank you, this is an important point. Our use of the term was not intended to imply that BSA-based dosing is inherently excessive, but that it can produce disproportionately higher absolute doses compared to weight-based approaches- we have changed the wording to make this clear in the final paragraph.
For aciclovir, the PK profile is characterised by renal elimination and a relatively wide therapeutic index in uncomplicated HSV infection. Aciclovir's volume of distribution correlates with IBW and total body water rather than total body weight (TBW), meaning BSA doses derived from TBW in children with obesity may exceed pharmacokinetically appropriate levels. In this context, efficacy is generally achieved at relatively modest plasma exposures, whereas toxicity becomes the main concern with increased doses, thus increasing the risk in those at higher body weight centiles.
Changes made: We have added a sentence on volume of distribution in the first paragraph for clarity of focus on potential overexposure rather than underdosing and corrected the wording of 'dose inflation'.

6) Your final paragraph about urgent reconsideration of existing guidelines and reducing avoidable harm isn't really supported with this single example. I think you have a reasonable call-to-action around re-evaluating dosing assumptions, but this requires further research.
Response: Reworded, thank you.
Changes made: The wording has been changed to reflect a call-to-action.

Reviewer: 2
Dr. Yue-E Wu, Shandong University
Comments to the Author
Overall, the article is concise and clinically relevant, and it raises an important issue regarding aciclovir dosing in children with obesity. However, I think there are still several points that need further clarification. My main concerns are as follows.

Major concerns
1. The current analysis appears to be based only on boys aged 0–17 years at the 50th height centile. Could the authors further discuss how generalisable these findings are to girls and to children with heights substantially different from the 50th centile?
Response: Thank you for this important point. The current analysis is based on boys at the 50th height centile for illustrative purposes, providing a simplified and reproducible reference population for exploring dose variation across weight and age. We recognise that this does not fully capture the range of anthropometric variability seen in clinical practice.
Extending the model to incorporate sex-specific growth standards and variation in height (e.g. across height SDS) represents a logical next step. This would allow a more comprehensive assessment of how differences in growth patterns and body proportions influence BSA estimation and downstream dosing.
However, given that BSA is fundamentally derived from height and weight, we would expect the key structural findings of the model, particularly the divergence between dosing approaches at extremes of body size, to remain directionally consistent, even if absolute dose values would vary.
Changes made: These limitations have now been acknowledged in the manuscript in the penultimate paragraph.

2. This work is essentially a guideline-based visualisation and theoretical modelling study, rather than a PK/PD or clinical outcome study. Is there any evidence that the proposed AdjBW-based BSA dosing

would maintain antiviral efficacy while reducing the risk of overdosing in children with obesity?
Response: Thank you for this important point. This work is a guideline-based visualisation and theoretical modelling study, and we acknowledge that there are no clinical or pharmacokinetic studies specifically validating our AdjBW-based BSA dosing for aciclovir in children with obesity. Our aim was to highlight the potential for disproportionate dose variation at extreme weight centiles and to explore whether a pragmatic adjustment could mitigate this effect.
From a pharmacokinetic perspective, aciclovir is predominantly renally cleared and distributes into lean body mass rather than adipose tissue. We have added a sentence about this in the first paragraph for clarity. As such, dosing based on total body weight may overestimate pharmacokinetically relevant distribution in children with obesity, whereas AdjBW offers a closer approximation of physiological scaling. In uncomplicated HSV infection, efficacy is achieved at standard exposures, with no clear evidence of improved outcomes at higher doses, while toxicity, particularly nephrotoxicity, is exposure-dependent.
We therefore present AdjBW-based BSA dosing as a pharmacokinetically informed approach to reducing the risk of overexposure. However, we acknowledge that its impact on antiviral efficacy and safety has not been directly evaluated, and these findings should be considered hypothesis-generating. Further work incorporating PK modelling and prospective clinical data will be required to determine whether this approach improves therapeutic outcomes.
Changes made: The lack of PK/ clinical data is mentioned in the penultimate paragraph alongside other limitations of our work.

3. The authors propose using AdjBW for children above the 50th centile weight-for-age. Why was the 50th centile selected as the threshold? Would it be more clinically appropriate to use an obesity-related threshold instead, such as a higher weight centile or BMI-based definition?
Response: Thank you for this important point. We agree that an obesity-related threshold, such as a higher weight centile or BMI-based definition, would be a clinically appropriate and potentially more practical criterion for implementing AdjBW in practice.
In this model, the 50th centile was selected as a reference point representing expected weight-for-age in a healthy population, allowing deviations in body composition to be explored systematically. The use of AdjBW above this point was intended to examine how progressively increasing divergence from expected weight influences dosing behaviour, rather than to define a binary clinical cut-off.
This approach enabled evaluation of dosing patterns across the full spectrum of weight centiles, capturing the gradient of divergence that begins below traditional obesity thresholds. However, we acknowledge that translating this into clinical practice would likely require a more clearly defined trigger, such as BMI-based obesity criteria.
Changes made: This point is included in the penultimate paragraph alongside other limitations of our work.

4. The manuscript uses the formula AdjBW = IBW + 0.35 × (Actual − IBW). Could the authors explain the rationale for choosing the 0.35 coefficient in this paediatric aciclovir context, and whether this coefficient has been validated for antiviral dosing in children?
Response: This coefficient was taken directly from official NPPG guidance (reference 6): ‘they recommend using an adjustment factor of 0.35 in most cases as a pragmatic solution to calculating adjusted body weight in children living with obesity’, as this would be the guidance used by most paediatric trusts in the country.
Changes made: No changes made as this is a direct citation.

5. The authors define IBW as the 50th centile BMI-for-age for the AdjBW calculation. Could they further justify why this particular definition of IBW was chosen, and whether other definitions of IBW might materially change the proposed dosing curves?
Response: Thank you for this important point. The 50th centile BMI-for-age definition of IBW was selected as it is the most widely used and operationally accessible reference in UK paediatric

practice, consistent with UKMi/NPPG guidance (reference 6). In addition, it provides a pragmatic approximation of expected body composition for age and sex, and therefore serves as a reasonable proxy for lean body mass when applying AdjBW in dosing calculations, particularly relevant for drugs such as aciclovir, where distribution and clearance are more closely related to lean mass and renal function than total body weight.
We acknowledge that alternative definitions of IBW (e.g. McLaren or Moore methods) could be used and would yield slightly different IBW estimates. However, these differences are unlikely to materially alter the overall shape of the dosing curves, as AdjBW is relatively insensitive to small variations in the IBW anchor, particularly given the 0.35 correction factor. Future work could incorporate sensitivity analyses comparing different IBW definitions to further quantify their impact on dosing outputs.
Changes made: Potential sensitivity analyses acknowledged in the limitations paragraph at the end of the manuscript.

6. To address the abrupt dose drop at age 12, the authors apply linear interpolation between BSA-based dosing and 5 mg/kg dosing from age 12 to 17 years. Why was a linear transition chosen, and why does the transition end specifically at 17 years? Would other transition functions or age ranges be more physiologically plausible?
Response: The transition was extended to 17 years to reflect the upper boundary of paediatric dosing in the BNFc, after which adult dosing strategies are typically applied.
The linear transition was chosen as a pragmatic and transparent method to smooth the discontinuity in dosing observed at age 12, rather than as a physiologically exact representation of drug handling. The underlying issue is that the BNFc introduces a step change from weight-based (mg/kg) to BSA-based (mg/m²) dosing at this age, which produces an abrupt and clinically implausible reduction in calculated dose in some patients. A linear interpolation was chosen for 3 main reasons:
1. Ensure a gradual and monotonic transition between the two dosing paradigms
2. Avoid introducing additional non-linear assumptions that are not clearly supported by pharmacokinetic data
3. Maintain interpretability and reproducibility within the model
Future work incorporating pharmacokinetic modelling may allow a more biologically grounded transition to be defined. However, in the absence of such data, and given the practical advantages of transparency and ease of implementation, a linear transition was chosen.
Changes made: No changes made as rationale explained, however the need for further clinical research and lack of PK data has been newly acknowledged in the penultimate paragraph.

7. The manuscript argues that the current BNFC approach leads to an abrupt drop from 570 mg at age 11 to 191.9 mg at age 12 in a very high weight-centile child, and that the proposed approach reduces the 11-year-old dose to 398.8 mg. However, how should readers interpret these numerical differences clinically, given that there are no linked exposure targets, toxicity thresholds, or virological outcomes presented?
Response: We appreciate that the clinical significance of the absolute dose differences cannot be directly interpreted in the absence of defined exposure- response relationships or clinical data. Our intention is not to propose a definitive target dose, but to highlight internal inconsistencies within the current prescription guidelines. The observed reduction from 570 mg at age 11 to 191.9 mg at age 12 in a high weight-centile child represents a marked discontinuity that is unlikely to reflect underlying pharmacokinetic or physiological changes. This may introduce unintended variability in exposure at the point of transition between dosing paradigms. The proposed model reduces this discontinuity by providing a more continuous relationship between body size and dose.
At the 99.99th centile, the reduction to 398.8 mg reflects the use of AdjBW generating a BSA that more closely approximates the pharmacokinetically relevant lean compartment. The clinical interpretation is not that 398.8 mg represents a validated target dose, but that dosing derived from a weight measure incorporating body composition is likely to be more physiologically defensible than one driven by total body weight alone in this group. We have incorporated a clause to address this in

the manuscript.
Changes made: A comment was added when describing Figure 1 to highlight the importance of AdjBW-BSA in approximating the lean compartment. Lack of PK/ clinical data has been added to the limitations.

8. Aciclovir exposure is influenced not only by body size, but also by other factors such as renal function, indication, and route of administration. Since the present analysis focuses mainly on anthropometric dosing rules, could the authors comment on how these additional determinants might affect the clinical applicability of the proposed model?
Response: We agree that aciclovir exposure is influenced by multiple factors beyond body size, including renal function, indication, and route of administration. These factors require independent clinical consideration and dose adjustment regardless of the underlying body weight metric used. The AdjBW approach therefore addresses only the anthropometric component of dosing, and should be viewed as complementary to these established clinical adjustments rather than a replacement for them.
Changes made: Acknowledged in the limitations paragraph alongside other factors.

Minor comments
9. The background states that conventional weight-based or BSA-based dosing may lead to underdosing or toxicity at the extremes of weight centiles. It would strengthen the letter if the authors could provide more direct references supporting this statement specifically in paediatric obesity or in aciclovir prescribing.
Response: Thank you. We have added reference 1 on the pharmacovigilance data from the MHRA Yellow Card Scheme specifically for aciclovir. We have already included some references discussing the challenges of paediatric prescribing in the context of obesity (e.g. references 3 and 6 in the bibliography) as well. However, we are constrained by both word count and reference limits for this letter, and we hope that future work will allow these issues to be explored in greater depth.
Changes made: Reference 1 added to the manuscript.

10. Some of the mathematical expressions in the proof version appear unclear or incompletely rendered, including the Boyd formula and the dose blending equation. I would suggest carefully checking the equation formatting in the final version. In addition, the phrase “AdjBW-base BSA dosing” in the figure legend may need correction to “AdjBW-based BSA dosing.”
Response: Thank you for this. The Boyd formula was taken directly from the manuscript of Sharkey et al. (2001) or reference 5, also quoted within the BNFc itself and we wanted this to remain as accurate as possible. The figure legend has been corrected and the equations formatted more homogenously.
Changes made: The figure legend has been corrected and the equations formatted more homogenously.
